# Hantavirus as a persistent zoonotic risk: epidemiology and current pharmacological management

**DOI:** 10.3389/fphar.2026.1925640

**Published:** 2026-09-18

**Authors:** Salvatore Di Maria, Alessio Provenzani

**Affiliations:** 1 School of Specialization in Hospital Pharmacy, University of Catania, Catania, Italy; 2 IRCCS ISMETT, Palermo, Italy; 3 UPMC Italy, Palermo, Italy

**Keywords:** Andes virus, companion animals, Hantavirus, hemorrhagic fever with renal syndrome, orthohantavirus, pharmacological treatment, ribavirin, solid organ transplantation

## Abstract

**Background:**

Hantaviruses are emerging rodent-borne zoonotic viruses that continue to pose a public health concern despite their relatively low incidence in humans. The 2026 *Andes virus* outbreak linked to the MV *Hondius* cruise ship renewed international attention on these pathogens, highlighting unresolved questions regarding transmission dynamics, vulnerable populations, and global preparedness. This review provides an updated critical appraisal of recent advances in hantavirus epidemiology, emerging therapeutic strategies, and public health challenges, with particular emphasis on the 2026 Andes virus outbreak, companion animals, and solid organ transplant recipients.

**Methods:**

A review of the recent literature was performed, focusing on zoonotic transmission, the role of companion animals, current and emerging therapeutic strategies, infections in immunocompromised and solid-organ transplant recipients, and the implications of the 2026 international outbreak.

**Results:**

Although the sentinel hypothesis remains plausible, companion animals are not natural reservoirs, and preliminary evidence suggests that their exposure may reflect local hantavirus circulation, while pet rats have emerged as an important source of *Seoul orthohantavirus* infection. Therapeutic options remain limited: supportive care is the standard of treatment, and ribavirin shows benefit only in early hemorrhagic fever with renal syndrome. Emerging immunotherapeutic approaches, including neutralizing monoclonal antibodies and host-directed therapies, show promising preclinical results but still lack robust clinical validation. In immunocompromised patients, particularly solid-organ transplant recipients, hantavirus infection may mimic graft rejection or present with atypical, potentially fatal manifestations, posing diagnostic challenges. The 2026 MV *Hondius* outbreak reinforced the need for heightened surveillance, rapid diagnosis, and coordinated international response, while confirming that sustained human-to-human transmission remains exceptional.

**Conclusion:**

Recent evidence broadens the clinical and public health relevance of hantaviruses beyond their traditional framing as rodent-borne zoonoses. Advances in targeted therapeutics, integrated surveillance, and One Health preparedness will be pivotal to improving outbreak preparedness and accelerating the development of effective preventive and therapeutic strategies.

## Graphical Abstract

**Figure:**
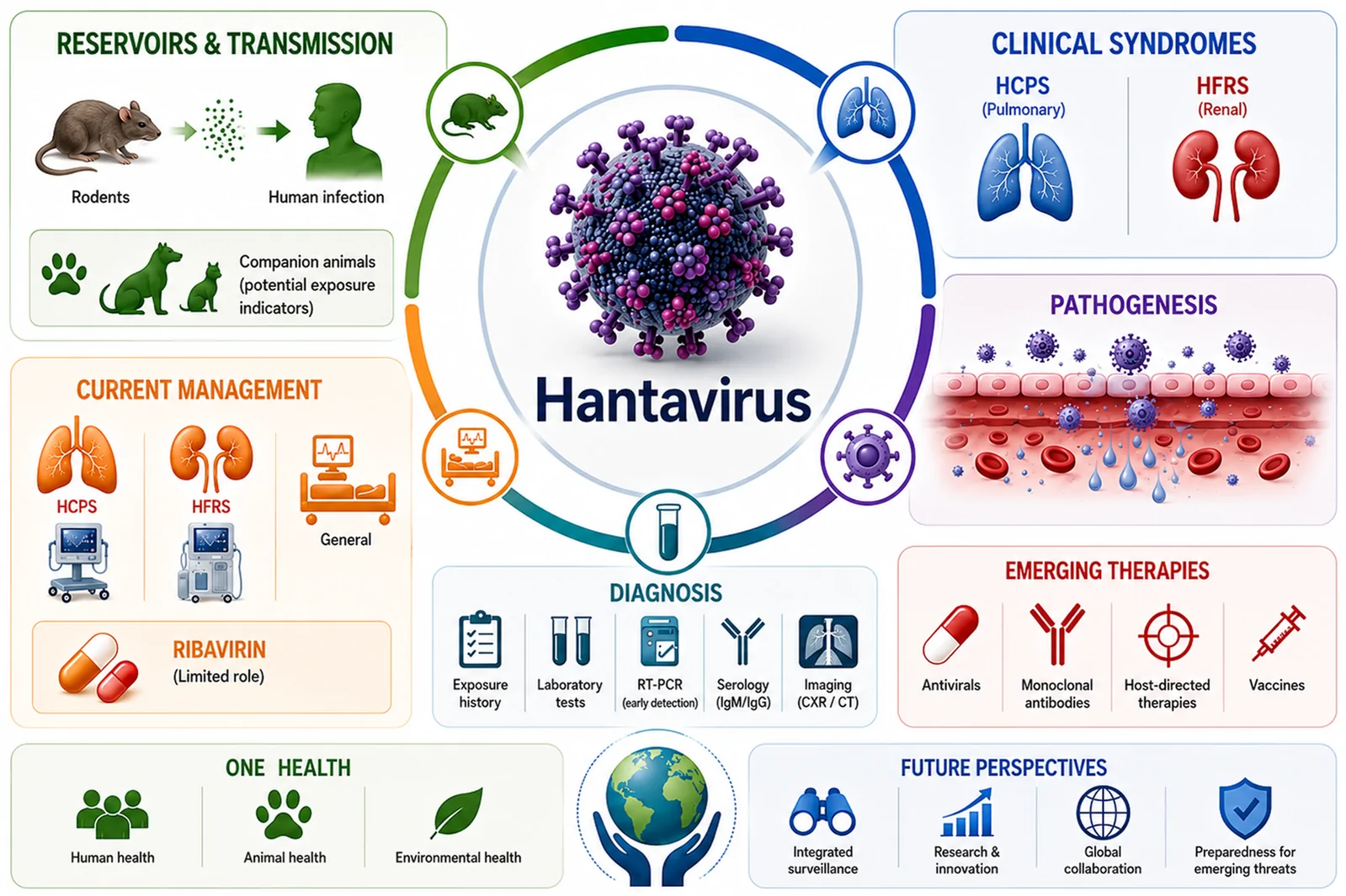

## 1 Introduction

Within the landscape of emerging infectious diseases, there are pathogens that, despite remaining far from the spotlight for long periods, continue to represent a constant source of uncertainty for global public health. Some of them emerge suddenly, spreading rapidly and placing considerable pressure on healthcare systems, economies, and societies worldwide. Others, however, follow a more discreet path: they persist silently within natural ecosystems, causing sporadic human infections and attracting only limited attention until an unexpected outbreak or a series of unusual epidemiological events brings their potential threat back to the attention of the scientific community and the media. Hantaviruses belong to this latter category. Despite the relative rarity of human infections, these zoonotic pathogens continue to raise questions regarding their true public health impact and the risk they may pose in the context of future infectious disease emergencies (Kumar and John, 2026). Nevertheless, despite substantial advances in hantavirus research, important knowledge gaps remain regarding their future epidemiological impact, the ecological drivers of spillover, and the development of effective therapeutic strategies.

Behind the relatively small number of human cases lies a biologically complex family of viruses. Hantaviruses belong to the family *Hantaviridae*, within the order *Bunyavirales*, and are enveloped viruses with a spherical morphology measuring approximately 80–120 nm in diameter. Their genome consists of a negative-sense single-stranded RNA segmented into three fragments, designated Small (S), Medium (M), and Large (L). The S segment encodes the nucleoprotein (N), which is involved in encapsidation of the viral genome; the M segment encodes the surface glycoproteins Gn and Gc, which are essential for host cell entry; whereas the L segment encodes the RNA-dependent RNA polymerase (RdRp), responsible for viral transcription and genome replication (Bradfute et al., 2024; Vaheri et al., 2013).

Replication occurs entirely within the cytoplasm of the infected cell. Following the binding of the viral glycoproteins to host cell receptors, the virus enters the cell by receptor-mediated endocytosis. After acidification of the endosome, the ribonucleocapsids are released into the cytoplasm, where the viral RNA polymerase initiates the synthesis of messenger RNAs and new genomic copies. Assembly of mature virions occurs predominantly within the Golgi apparatus, from which newly formed viral particles are subsequently released (Vaheri et al., 2013; Vial et al., 2023).

The apparent genomic simplicity of hantaviruses should not be misleading. Like many RNA viruses, they possess remarkable evolutionary plasticity, largely due to the lack of efficient proofreading mechanisms during genome replication. The progressive accumulation of mutations, reassortment between different genomic segments, and adaptation to novel reservoir hosts have contributed over time to the considerable genetic diversity observed within the *Hantaviridae* family. Although most of these genetic changes do not necessarily translate into increased transmissibility or virulence, the continuous evolution of hantaviruses remains one of the main factors driving scientific interest in these pathogens and their emerging potential (Vial et al., 2023; Klempa, 2018).

The history of infectious diseases has repeatedly demonstrated that many epidemics and pandemics have originated from spillover events, namely, the transmission of pathogens from their animal reservoirs to humans. One emblematic example is plague caused by *Yersinia pestis*, the agent responsible for the Black Death in the 14th century and for the subsequent epidemic that struck the Duchy of Milan between 1629 and 1631, famously described by Alessandro Manzoni in *The Betrothed* (Plo et al., 2017). Likewise, hantaviruses represent a paradigmatic example of an emerging zoonosis. Their natural reservoirs consist predominantly of wild rodents, which harbor the virus persistently and generally remain asymptomatic. Human infection occurs accidently through the inhalation of aerosols contaminated with infected urine, saliva, or feces, without the need for direct contact with the animal (Tortosa et al., 2024; Tian et al., 2017).

The public health risk associated with hantaviruses therefore stems not primarily from their ability to sustain human-to-human transmission chains, but rather from the continuous exposure of humans to ecosystems in which the virus naturally circulates. Climate change, habitat fragmentation, deforestation, and the expansion of human activities into rural and peri-urban environments are progressively increasing opportunities for contact between humans and wildlife, thereby creating favorable conditions for new spillover events and the emergence of unexpected outbreaks (Fabbri et al., 2025).

Recent evidence has further highlighted how climate change, biodiversity loss, and land-use modifications are reshaping pathogen ecology and increasing opportunities for zoonotic spillover, reinforcing concerns regarding the future emergence of hantavirus outbreaks and other emerging zoonoses (Carlson et al., 2025).

As a consequence, a genuine environmental pressure is reshaping human exposure to these pathogens through changing ecosystem dynamics.

However, unlike highly transmissible respiratory viruses, hantaviruses generally do not sustain human-to-human transmission (Toledo et al., 2022). Within the biological and virological diversity of hantaviruses (Hepojoki et al., 2015), Andes virus (ANDV) represents a notable epidemiological exception, as person-to-person transmission has been documented under specific conditions, particularly following close and prolonged contact (World Health Organization, 2026a). Epidemiological and virological studies from South America have documented transmission within households and healthcare settings and characterized viral shedding during acute infection (Martinez-Valdebenito et al., 2014; Ferrés et al., 2024). A recent systematic evidence assessment further indicated that documented person-to-person transmission is restricted to ANDV, with no evidence of asymptomatic or presymptomatic transmission (UK Health Security Agency, 2026). Although transmission clusters and occasional superspreading events have been reported under specific circumstances (Martínez et al., 2020), the available evidence does not support sustained transmission within the general population. Beyond the unresolved epidemiological questions, major uncertainties also persist regarding the therapeutic management of hantavirus infection. Clinical care remains largely supportive, while no antiviral therapy has yet demonstrated consistent clinical benefit in adequately powered randomized trials. Recent advances in antiviral discovery, including computational drug design, natural product-based approaches, targeted drug delivery, and host-directed therapeutic strategies, have renewed interest in the development of novel interventions for hantavirus infection. Accordingly, investigational approaches including direct-acting antivirals, broadly neutralizing monoclonal antibodies, immune-based interventions, and host-directed therapies have shown encouraging activity in experimental models and early clinical studies; however, no pharmacological intervention has yet demonstrated unequivocal efficacy in randomized clinical trials, and the available evidence remains fragmented. These gaps underscore the need for well-designed clinical trials to establish the safety and efficacy of emerging antiviral agents, monoclonal antibodies, and host-directed therapeutic strategies, which remain key priorities for future research (Martin and Tripp, 2024; Afzal et al., 2023; Lanrewaju et al., 2026).

So, is hantavirus merely a flash in the pan, or an emerging threat? The currently available evidence suggests that the answer depends less on the limited human-to-human transmission observed today than on the ability of these viruses to persist within their natural reservoirs, adapt to ecological changes, and continuously generate new opportunities for spillover (Provenzani and Polidori, 2020). From this perspective, hantaviruses do not represent an imminent pandemic threat; rather, they constitute a paradigmatic example of a persistent zoonotic risk that requires continuous epidemiological surveillance, clinical preparedness, and an integrated One Health approach.

Although previous reviews have comprehensively summarized hantavirus biology, epidemiology, and clinical manifestations, recent advances in therapeutic research warrant an updated critical appraisal of emerging pharmacological strategies. Accordingly, this review integrates current evidence on antiviral agents, monoclonal antibodies, immune-based interventions, and host-directed therapies while highlighting the principal unmet clinical needs and priorities for future research.

## 2 Methods

This work was conducted as a narrative, non-systematic review aimed at providing an updated overview of the epidemiology, clinical management, emerging therapeutic strategies, and public health implications of hantavirus infections. A comprehensive literature search was performed using PubMed/MEDLINE as the primary biomedical database. Searches combined free-text terms and, where appropriate, Medical Subject Headings (MeSH) using Boolean operators (AND, OR). Search terms included “hantavirus”, “hemorrhagic fever with renal syndrome”, “hantavirus pulmonary syndrome”, “Andes virus”, “Seoul virus”, “kidney transplantation”, and “companion animals”. Additional searches were conducted in ClinicalTrials.gov to identify ongoing and completed clinical trials related to hantavirus therapeutics and vaccines.

To incorporate the most recent epidemiological evidence and international public health recommendations, additional information was retrieved from authoritative institutional sources, including the World Health Organization (WHO), the Centers for Disease Control and Prevention (CDC), the UK Health Security Agency (UKHSA), and the Istituto Superiore di Sanità (ISS). Particular attention was devoted to the WHO Disease Outbreak News reports, the Hantavirus Outbreak Toolbox, and the interim guidance documents published following the 2026 Andes virus outbreak associated with the MV *Hondius* expedition.

The literature search covered publications from database inception through June 2026. Eligible sources included narrative and systematic reviews, randomized controlled trials, observational studies, prospective and retrospective cohort studies, case reports, case series, clinical practice guidance, outbreak investigation reports, and official documents issued by international public health agencies. Reference lists of the selected publications were also manually screened to identify additional relevant studies. Evidence was critically appraised and synthesized to provide a balanced overview of the current knowledge regarding hantavirus epidemiology, zoonotic transmission, pharmacological management, emerging therapeutic approaches, infections in immunocompromised and solid organ transplant recipients, and the implications of recent international outbreaks. Owing to the narrative design of this review, no quantitative synthesis or meta-analysis was performed.

## 3 Hantavirus and companion animals

Although hantaviruses have traditionally been regarded as pathogens associated with wild rodents, growing evidence suggests that companion animals may also be exposed to these viruses and, in some cases, contribute to their epidemiology (Canadian Paediatric Society, 2000; Dobly et al., 2012). A seroepidemiological study conducted in Belgium involving 410 dogs and 124 domestic cats detected anti-hantavirus antibodies in both species, revealing a significantly higher seroprevalence in cats, the natural predators of rodents, than in dogs (16.9% vs. 4.9%; *p* = 0.001) (Dobly et al., 2012). Furthermore, dogs living in southern Belgium, a region characterized by greater forest cover and a higher rodent density, showed significantly higher seropositivity rates than those from the northern part of the country (10.5% vs. 3.0%; *p* = 0.002). Similarly, seropositive cats were more frequently found in highly forested areas than seronegative animals. These findings suggest that exposure to hantaviruses in dogs and cats may reflect the ecological patterns of local viral circulation observed in human populations; however, the sentinel hypothesis remains plausible and requires further validation.

Over time, attention has progressively shifted toward the role of domestic rodents in the zoonotic transmission of hantaviruses. In particular, *Seoul orthohantavirus* (SEOV), a hantavirus primarily associated with the brown rat (*Rattus norvegicus*), has been implicated in numerous human cases reported in Europe and North America, frequently linked to contact with pet rats or rats bred as feeder animals (Heuser et al., 2023). Several studies have documented a high prevalence of infection among domestic rats, together with cases of hemorrhagic fever with renal syndrome (HFRS) occurring in pet owners and breeders, highlighting SEOV as an emerging public health concern (Clement et al., 2019; Rubin, 2017; McElhinney et al., 2017).

Although human infection often follows a mild or atypical clinical course, the increasing number of reported cases underscores the need to strengthen epidemiological surveillance of companion rodents belonging to the families Muridae and Cricetidae. It also emphasizes the importance of increasing awareness among both clinicians and veterinarians regarding the risk of zoonotic transmission associated with these animals (Table 1).

**TABLE 1:** One health perspective on hantavirus transmission.

Component	Current evidence	Clinical and public health relevance
Wild rodents	Primary reservoir for all pathogenic hantaviruses	Main driver of zoonotic spillover
Companion dogs	Serological markers of environmental exposure	Exposure may reflect local viral circulation
Companion cats	Higher seroprevalence than dogs due to rodent predation	Exposure may reflect local viral circulation
Pet rats	Reservoir of SEOV associated with documented human outbreaks	Emerging domestic zoonotic risk
Environmental changes	Climate change, urbanization and habitat fragmentation increase rodent–human contact	Increased probability of spillover events
Integrated surveillance	Human, veterinary and environmental monitoring	Cornerstone of One Health preparedness

This need becomes even more relevant in light of the growing presence of synanthropic rodents in urban environments, a phenomenon that is particularly evident during the warmer months. Following the breeding season, rodent populations increase substantially, leading to the colonization of storm drains, waste disposal areas, and, not infrequently, private homes, kitchens, warehouses, and food preparation or storage facilities, thereby increasing opportunities for human exposure.

## 4 Current status and future directions of pharmacological management

At present, no specific antiviral therapy has been approved for the treatment of hantavirus infections. Clinical management is therefore largely supportive and depends on the clinical syndrome developed by the patient, which is determined by the infecting viral species (Table 2). The two major clinical manifestations, Hantavirus Pulmonary Syndrome (HPS), also referred to as Hantavirus Cardiopulmonary Syndrome (HCPS), and Hemorrhagic Fever with Renal Syndrome (HFRS), differ substantially in their pathophysiology and consequently require distinct therapeutic approaches. Old World hantaviruses, such as Hantaan and Puumala viruses, predominantly cause HFRS, whereas New World hantaviruses, including Sin Nombre virus and Andes virus, are mainly associated with HPS/HCPS.

**TABLE 2:** Current and emerging therapeutic strategies for hantavirus infection.

Therapeutic strategy	Mechanism of action	Clinical evidence	Current status/Limitations
Supportive care	Respiratory, hemodynamic and renal support	Standard treatment for both HFRS and HCPS	Remains the cornerstone of management; no antiviral effect
Ribavirin	Inhibits viral RNA synthesis through RdRp inhibition, IMPDH inhibition and induction of lethal mutagenesis	Demonstrated benefit only when administered early in HFRS, particularly in hantaan virus infection	Ineffective in HCPS; requires early diagnosis; off-label (not FDA/EMA approved)
Corticosteroids	Broad suppression of the inflammatory response	Randomized clinical trial failed to demonstrate improvement in mortality or clinical outcomes	Not recommended for routine treatment
Empirical antibiotics	Treatment of possible bacterial co-infection	Frequently initiated during the prodromal phase because of nonspecific presentation	Should be discontinued once bacterial infection is excluded
Vasopressors (norepinephrine ± dobutamine)	Hemodynamic stabilization in shock	Recommended in severe HCPS requiring intensive care	Supportive therapy only
Renal replacement therapy	Management of acute kidney injury	Indicated in severe HFRS with renal failure	Organ support without antiviral activity
Favipiravir, ETAR, baloxavir	Direct inhibition of viral replication	Promising results in experimental models	Human efficacy remains unproven
Host-directed therapies (vandetanib, icatibant)	Target endothelial dysfunction and vascular leakage	Encouraging preclinical findings	Clinical studies are still lacking
Neutralizing monoclonal antibodies	Block viral entry by targeting Gn/Gc glycoproteins	High efficacy in animal models	Not yet available for clinical use
DNA vaccines	Induction of virus-specific neutralizing antibodies	Phase I–II clinical trials demonstrate safety and immunogenicity	No licensed vaccine currently available

In patients with Hantavirus Pulmonary Syndrome (HPS), the rapid progression to respiratory failure necessitates prompt hospitalization, often in an intensive care unit. Clinical management relies on continuous monitoring of cardiovascular and respiratory function, with particular attention to maintaining adequate hemodynamic stability. Fluid administration should be carefully titrated because of the pulmonary edema resulting from the increased capillary permeability that characterizes the disease. Supplemental oxygen is indicated in patients with hypoxemia, while mechanical ventilation is required in more severe cases. In patients with refractory respiratory failure, highly specialized centers may consider extracorporeal membrane oxygenation (ECMO), which has been shown to improve survival in selected patients.

Because the initial symptoms of HPS are nonspecific and may mimic severe bacterial infection, early empirical treatment with broad-spectrum antibiotics is recommended until alternative infectious etiologies have been excluded. Management also includes symptomatic supportive measures, such as antipyretics and analgesics to control fever and pain. As the disease progresses, hypotension is frequently accompanied by worsening pulmonary edema and severe hypoxemia, often requiring invasive ventilatory support. Among the major adverse prognostic factors identified in patients with HPS are a plasma lactate concentration greater than 4.0 mmol/L and a cardiac index below 2.2 L/min/m^2^, both of which reflect severe hemodynamic compromise and are associated with an increased risk of mortality (Centers for Disease Control and Prevention, 2026).

In contrast, Hemorrhagic Fever with Renal Syndrome (HFRS) exhibits a highly variable clinical course that is closely related to the infecting hantavirus species. Infections caused by Hantaan virus and Dobrava–Belgrade virus are generally associated with more severe disease, whereas those caused by Seoul, Saaremaa, and Puumala viruses usually present with milder clinical manifestations. The incubation period typically ranges from one to 2 weeks after viral exposure. Disease onset is usually abrupt and characterized by high fever, severe headache, lumbar and abdominal pain, chills, nausea, and visual disturbances, often accompanied by facial flushing, conjunctival injection, and skin rash. As the disease progresses, patients may develop hypotension, thrombocytopenia, impaired renal function, oliguria, and, in severe cases, acute kidney injury. Although their clinical manifestations differ, HPS and HFRS share a common pathogenic mechanism, namely, increased endothelial permeability resulting in widespread vascular dysfunction.

As with HPS, no specific etiological treatment is currently available for HFRS. Management is therefore primarily supportive and aimed at preventing or limiting systemic complications. Clinical care includes careful monitoring of fluid balance and electrolyte status, maintenance of adequate oxygenation and blood pressure, and treatment of secondary bacterial infections when present. In patients who develop severe acute kidney injury, renal replacement therapy, including dialysis, may be required to correct fluid overload, electrolyte disturbances, and the accumulation of nitrogenous waste products, representing a key intervention for improving clinical outcomes (Centers for Disease Control and Prevention, 2024).

### 4.1 Pharmacological therapies evaluated in clinical practice

#### 4.1.1 Ribavirin

To date, ribavirin is the most extensively investigated antiviral agent for the treatment of hantavirus infections and remains the only drug that has demonstrated a clinical benefit in humans, although this benefit is limited to selected forms of Hemorrhagic Fever with Renal Syndrome (HFRS). Ribavirin is a synthetic guanosine analogue with broad-spectrum antiviral activity against numerous RNA and DNA viruses. Its mechanism of action is multifactorial and includes inhibition of inosine monophosphate dehydrogenase (IMPDH), resulting in depletion of intracellular guanosine triphosphate (GTP) pools, inhibition of the viral RNA-dependent RNA polymerase (RdRp), interference with viral RNA capping, and induction of an increased mutation rate within the viral genome (“error catastrophe”), ultimately leading to the production of nonviable viral particles (Graci and Cameron; Nyström et al., 2019).

The efficacy of ribavirin is highly dependent on the timing of treatment initiation. Because its antiviral activity primarily targets the viral replication phase, therapy should be started during the early stages of infection, before the onset of the systemic immune response responsible for endothelial dysfunction and increased vascular permeability, which are the main determinants of disease severity. This represents one of the major limitations of ribavirin in clinical practice, as the diagnosis is frequently established only after immunopathological complications have become the predominant feature of the disease rather than active viral replication (Klempa, 2018).

The strongest clinical evidence derives from a prospective, randomized, double-blind, placebo-controlled trial conducted by Huggins et al. involving 242 patients with serologically confirmed HFRS in China (Huggins et al., 1991). In this study, ribavirin was administered intravenously using a loading dose of 33 mg/kg, followed by 16 mg/kg every 6 h for 4 days and subsequently 8 mg/kg every 8 h for an additional 3 days. Treatment resulted in a significant reduction in mortality, with an approximately seven-fold lower risk of death compared with placebo after adjustment for major prognostic factors. In addition, treated patients were less likely to develop the oliguric phase and the hemorrhagic complications characteristic of HFRS. The only treatment-related adverse event was reversible hemolytic anemia, consistent with the well-established safety profile of ribavirin.

A subsequent meta-analysis by Moreli et al., which included both available clinical studies and the main preclinical evidence, confirmed that the efficacy of ribavirin is strongly influenced by both the timing of treatment initiation and the infecting hantavirus species (Moreli et al., 2014). In humans, clinical benefit has been demonstrated primarily in early HFRS caused by Hantaan virus (HTNV), where prompt administration of ribavirin was associated with reduced mortality and a lower risk of progression to severe disease. In contrast, evidence remains much more limited for infections caused by Dobrava–Belgrade virus (DOBV), while no significant benefit has been demonstrated in infections due to Puumala virus (PUUV) or in HPS/HCPS caused by New World hantaviruses, including Sin Nombre virus (SNV) and Andes virus (ANDV). The authors emphasized that these conclusions should be interpreted with caution because of the limited number of clinical studies and the considerable heterogeneity of the available evidence. Overall, the meta-analysis supports the use of ribavirin only in the early stages of HFRS, particularly in infections caused by HTNV, whereas the available data do not support its routine use in PUUV infection or in HPS/HCPS.

Current recommendations from the Centers for Disease Control and Prevention (CDC) suggest that ribavirin may be considered in patients with severe HFRS when the diagnosis is established early, while emphasizing that the available evidence remains limited and has not resulted in regulatory approval for this indication (Centers for Disease Control and Prevention, 2024).

The situation is different for Hantavirus Pulmonary Syndrome (HPS/HCPS), in which ribavirin has failed to demonstrate clinically meaningful benefit (Centers for Disease Control and Prevention, 2026). Studies involving patients infected with Andes virus or Sin Nombre virus have shown no reduction in mortality or improvement in respiratory outcomes. This lack of efficacy is likely explained by the unique pathophysiology of HPS, in which the symptomatic phase coincides with an intense host immune response characterized by endothelial dysfunction, release of pro-inflammatory cytokines, and severe capillary leak syndrome. In this setting, inhibition of viral replication alone is insufficient to alter disease progression once immune-mediated tissue damage has been established.

From a regulatory perspective, ribavirin has not been approved by either the U.S. Food and Drug Administration (FDA) or the European Medicines Agency (EMA) for the treatment of hantavirus infections, and its use remains off-label. Nevertheless, it remains the only antiviral agent for which clinical efficacy has been demonstrated in humans, albeit exclusively in early HFRS. The absence of documented benefit in HPS and the strict dependence on early treatment initiation underscore the need for novel antiviral agents capable of retaining efficacy during later stages of disease, as well as combination strategies that integrate inhibition of viral replication with modulation of the host immune response.

#### 4.1.2 Immunomodulation: Corticosteroids

The central role of the host immune response in the pathogenesis of hantavirus infections has provided the main rationale for evaluating corticosteroids as adjunctive therapy. Activation of immune cells and the release of pro-inflammatory cytokines, including tumor necrosis factor-α (TNF-α) and interleukin-6 (IL-6), contribute to endothelial dysfunction and increased vascular permeability, which represent key pathogenic mechanisms underlying both HFRS and HPS/HCPS. Based on these observations, it has been hypothesized that pharmacological modulation of the inflammatory response could reduce endothelial injury and improve clinical outcomes.

The strongest clinical evidence comes from the multicenter, randomized, double-blind, placebo-controlled trial conducted by Vial et al. (NCT00128180) (Vial et al., 2013), which evaluated high-dose intravenous methylprednisolone (16 mg/kg/day, up to a maximum of 1 g/day, for three consecutive days) in patients with Andes virus (ANDV)-associated HCPS. The study, performed across 13 centers in Chile, demonstrated no significant differences between the methylprednisolone and placebo groups with respect to either the composite primary endpoint or mortality. Although methylprednisolone showed a favorable safety profile, with no increase in viral load or treatment-related adverse events, no clinically meaningful therapeutic benefit was observed.

The lack of efficacy suggests that nonspecific immunosuppression alone is insufficient to modify disease progression. Endothelial dysfunction in hantavirus infection results from the interaction of multiple pathogenic mechanisms, including complement activation, dysregulation of the VEGF/VEGFR2 signaling pathway, bradykinin-mediated vascular leakage, and cell-mediated immune responses. Consequently, corticosteroids are not currently recommended for the routine treatment of hantavirus infections, and their use should be restricted to concomitant indications unrelated to the viral infection itself (Bi et al., 2008; Ulloa-Morrison et al., 2024). The negative findings of this trial have redirected research toward more selective immunomodulatory strategies targeting specific pathways involved in endothelial barrier disruption rather than broad suppression of the inflammatory response.

#### 4.1.3 Other supportive pharmacological treatments

In the absence of approved antiviral therapies, the pharmacological management of hantavirus infections remains largely supportive and complements the intensive care measures described above. Because the prodromal phase is characterized by nonspecific symptoms that may resemble bacterial sepsis or community-acquired pneumonia, empirical treatment with broad-spectrum antibiotics is frequently initiated during the early stages of illness. Antibiotic therapy should subsequently be reassessed and discontinued once hantavirus infection has been confirmed and concomitant bacterial infection has been excluded (Ulloa-Morrison et al., 2024; Dheerasekara et al., 2020).

At the same time, antipyretic and analgesic agents, particularly acetaminophen (paracetamol), are commonly administered to relieve fever, myalgia, and headache, thereby improving patient comfort (Munir et al., 2021). In patients with HPS/HCPS, the development of hypotension and shock frequently necessitates vasopressor therapy, with norepinephrine considered the first-line agent for maintaining adequate systemic perfusion. In cases complicated by myocardial dysfunction, dobutamine may be added to improve cardiac output and optimize hemodynamic support. Fluid therapy should be carefully individualized to avoid exacerbating pulmonary edema secondary to the increased capillary permeability that characterizes the disease (Ulloa-Morrison et al., 2024).

In HFRS complicated by severe acute kidney injury, pharmacological management is often combined with renal replacement therapy, including intermittent hemodialysis or continuous renal replacement therapy (CRRT), when indicated to control fluid overload, electrolyte disturbances, and uremia (Dheerasekara et al., 2020; Munir et al., 2021). Although none of these interventions directly targets viral replication, they remain the cornerstone of clinical management, providing vital organ support until the acute phase of the disease resolves.

### 4.2 Future directions

Despite significant advances in understanding the pathogenesis of hantavirus infections, no specific antiviral therapy has yet been approved, highlighting the need for the development of innovative pharmacological strategies. Future therapeutic approaches are increasingly focused on a multimodal strategy that combines direct-acting antivirals, immunotherapy, and host-directed therapies, with the aim of targeting both viral replication and the mechanisms responsible for endothelial dysfunction and increased vascular permeability, the principal determinants of disease severity (Munir et al., 2021; Liu et al., 2020).

Passive immunotherapy represents one of the first biological strategies investigated in humans and provides the conceptual bridge toward the development of antibody-based therapies. A multicenter clinical study conducted in Chile showed that the administration of immune plasma obtained from Andes virus (ANDV) convalescent patients was well tolerated and associated with encouraging signals of clinical benefit in patients with hantavirus cardiopulmonary syndrome (HCPS), suggesting that early administration of neutralizing antibodies may favorably influence disease progression. Although these findings require confirmation in randomized controlled trials, they provided the proof-of-concept that neutralizing antibodies can represent an effective therapeutic strategy and established the biological rationale for the subsequent development of recombinant monoclonal antibodies directed against hantaviruses (Vial et al., 2015; Brocato and Hooper, 2019).

Among the antiviral agents currently under investigation, favipiravir is considered the most promising candidate. This nucleoside analogue, which inhibits the viral RNA-dependent RNA polymerase (RdRp), has demonstrated potent antiviral activity against several hantavirus species in experimental models, reducing viral replication and improving survival when administered during the early stages of infection. Other compounds, including 1-β-D-ribofuranosyl-3-ethynyl- (Kumar and John, 2026; Bradfute et al., 2024; Vial et al., 2023)triazole (ETAR) and baloxavir, have also shown encouraging results in preclinical studies; however, clinical evidence supporting their use in humans remains insufficient (Munir et al., 2021; Liu et al., 2020; Paludan and Cherry, 2026).

At the same time, increasing attention has been directed toward host-directed therapies, which aim to mitigate endothelial injury rather than directly inhibit viral replication. In particular, inhibition of the vascular endothelial growth factor (VEGF)/VEGF receptor 2 (VEGFR2) signaling pathway with vandetanib and blockade of the bradykinin pathway with icatibant have shown promising results in experimental models, suggesting a potential role in limiting the vascular leakage that characterizes both HPS/HCPS and HFRS. Nevertheless, these approaches remain at the preclinical stage or are supported only by limited clinical experience, underscoring the need for well-designed prospective clinical trials to establish their efficacy and safety (Munir et al., 2021; Liu et al., 2020).

Building upon the proof-of-concept provided by convalescent immune plasma, passive immunotherapy has subsequently evolved toward the development of neutralizing monoclonal antibodies targeting the viral glycoproteins Gn and Gc. Several recombinant monoclonal antibodies have demonstrated potent neutralizing activity and protective efficacy in animal models of Andes virus (ANDV) infection, making this one of the most promising biological strategies currently under development.

Recent advances have substantially strengthened the rationale for monoclonal antibody-based therapies. Initial preclinical studies demonstrated that neutralizing monoclonal antibodies directed against the Gn and Gc glycoproteins provide robust protection in animal models of Andes virus infection, even when administered after viral exposure (Duehr et al., 2020). More recently, high-resolution structural studies have identified conserved neutralizing epitopes within the hantavirus glycoprotein spike complex, providing a rational basis for the development of broadly reactive monoclonal antibodies (Guo et al., 2026). These findings support the transition from proof-of-concept studies toward the rational design of next-generation immunotherapeutic agents with broader antiviral activity (Ramos-Nino et al., 2026).

However, no monoclonal antibody has yet progressed to advanced clinical trials or received regulatory approval for clinical use (Liu et al., 2020; Brocato and Hooper, 2019; Paludan and Cherry, 2026).

Vaccine development has also made substantial progress in recent years. The most advanced candidates are DNA vaccines designed to elicit neutralizing antibodies against the surface glycoproteins of hantaviruses. A phase IIa clinical trial evaluating a bivalent Hantaan virus/Puumala virus (HTNV/PUUV) DNA vaccine delivered by intramuscular electroporation demonstrated a favorable safety profile and robust immunogenicity, supporting the continued clinical development of this vaccine platform (Hooper et al., 2020). Similarly, a more recent phase I clinical trial of a DNA vaccine targeting Andes virus (ANDV) confirmed its safety, tolerability, and ability to induce a strong neutralizing antibody response in healthy volunteers (Paulsen et al., 2024). In parallel, additional clinical trials evaluating DNA vaccines against HTNV, PUUV, and ANDV have been registered on ClinicalTrials.gov (NCT02116205, NCT02776761, and NCT03682107), reflecting the growing interest in preventive strategies aimed at reducing the risk of infection among high-risk populations. Despite these encouraging findings, no vaccine has yet been approved by either the U.S. Food and Drug Administration (FDA) or the European Medicines Agency (EMA) for the prevention of hantavirus infections (Liu et al., 2020; Paulsen et al., 2024).

Overall, future therapeutic strategies are increasingly moving toward a pathogenesis-driven approach, in which direct-acting antivirals, immunotherapy, and host-directed therapies are implemented according to the stage of disease. Such an approach has the potential to provide a more personalized management of hantavirus infections by simultaneously targeting viral replication and the host mechanisms responsible for endothelial dysfunction and vascular leakage, thereby overcoming the current limitations of supportive care alone (Paludan and Cherry, 2026).

## 5 Hantavirus infection in immunocompromised patients: Implications for solid organ transplantation

The diagnosis of hantavirus infection is often challenging and delayed because of the nonspecific clinical manifestations that characterize the early stages of the disease. Although the current epidemiological situation appears relatively stable, hantaviruses continue to represent an important public health concern. Their prolonged incubation period, which may extend up to 42 days depending on the viral species, allows new cases to be identified several weeks after exposure. In this context, preventive measures play a crucial role and should target the general population, with particular attention to individuals at increased risk of exposure, including healthcare workers, personnel employed in rodent-infested environments such as waste management workers, pest control operators, agricultural and forestry workers, as well as vulnerable populations, including immunocompromised patients and solid organ transplant recipients, in whom infection may follow a more severe clinical course.

Among transplant recipients, only a limited number of hantavirus infections have been reported in the literature, and, to date, no cases specifically associated with Andes virus have been described. Nevertheless, maintaining a high level of clinical suspicion for emerging infections remains essential in immunocompromised patients, particularly when pathogens with potential respiratory transmission are involved. Although donor-derived transmission through solid organ transplantation is not currently considered a significant concern, careful assessment of epidemiological exposures, including travel history and participation in community settings associated with an increased risk of infection, should be incorporated into the evaluation of both transplant candidates and recipients (Table 3).

**TABLE 3:** Hantavirus infection in solid organ transplant recipients: why it matters for transplant physicians.

Clinical challenge	Evidence from current literature	Implications for transplant practice
Low clinical suspicion	Only sporadic cases have been reported, making hantavirus an often-overlooked diagnosis in transplant recipients	Maintain a high index of suspicion in patients with compatible epidemiological exposure
Atypical clinical presentation	Immunosuppression may modify or attenuate the classic manifestations of HFRS or HCPS	Clinical presentation may be misleading, delaying diagnosis
Acute graft dysfunction	Hantavirus nephropathy may closely resemble acute cellular rejection, both clinically and histologically	Avoid unnecessary escalation of immunosuppressive therapy before excluding viral infection
Histopathological overlap	Tubulointerstitial nephritis and hemorrhagic lesions may mimic banff rejection	Correlation between pathology, laboratory findings and epidemiological history is essential
Broad differential diagnosis	Clinical features overlap with BK virus nephropathy, CMV infection, calcineurin inhibitor toxicity and bacterial sepsis	Hantavirus should be included in the diagnostic algorithm of unexplained graft dysfunction
Neurological involvement	Rare cases of fatal CNS infection have recently been reported in kidney transplant recipients	Consider hantavirus in unexplained encephalitis, particularly when conventional microbiological tests are negative
Diagnostic strategy	Serology and RT-PCR remain first-line tests; mNGS may be useful in selected complex cases	Early laboratory confirmation may prevent inappropriate management
Therapeutic management	Treatment remains largely supportive; no transplant-specific antiviral therapy is available	Management should combine supportive care with individualized adjustment of immunosuppression
Emerging relevance	The 2026 MV *Hondius* outbreak has renewed international awareness of hantavirus infection and highlighted the importance of rapid recognition of emerging zoonoses	Transplant physicians should remain vigilant, particularly in patients with recent travel history or relevant environmental exposure

Accordingly, hantavirus infection should be included in the differential diagnosis of transplant recipients presenting with fever, acute respiratory illness, or unexplained acute kidney injury (AKI), particularly following recent international travel or relevant environmental exposure (Table 3). This is especially important because hantavirus-associated nephropathy may clinically and histopathologically mimic acute allograft rejection, potentially leading to inappropriate intensification of immunosuppressive therapy. The diagnostic work-up should primarily include serological testing for anti-hantavirus IgM and IgG antibodies, molecular assays such as reverse transcription polymerase chain reaction (RT-PCR), and, in selected cases, immunohistochemistry or metagenomic next-generation sequencing (mNGS) when conventional diagnostic approaches fail to establish the diagnosis (Pereira, 2026).

Among kidney transplant recipients, hantavirus infection represents an exceptionally rare cause of acute kidney injury (AKI), in whom the differential diagnosis of graft dysfunction is generally focused on more common etiologies, including acute rejection, calcineurin inhibitor nephrotoxicity, and opportunistic infections. Indeed, viral infections are a well-recognized cause of acute renal allograft dysfunction in immunosuppressed patients, most commonly resulting from cytomegalovirus (CMV) or BK polyomavirus infection, which may lead to acute rejection, interstitial nephritis, or ureteral stenosis. However, acute kidney injury is also the hallmark organ manifestation of hemorrhagic fever with renal syndrome (HFRS) caused by hantaviruses in Europe and Asia, with disease severity varying according to the infecting viral species. In Central Europe, infections caused by Puumala virus (PUUV) and Dobrava–Belgrade virus (DOBV) usually present as mild-to-moderate forms of HFRS and are generally associated with complete recovery of renal function. Nevertheless, renal biopsy specimens obtained during the acute phase typically demonstrate acute tubulointerstitial nephritis, which may closely resemble acute allograft rejection on histopathological examination.

This diagnostic challenge was illustrated by the case of an adult living-donor kidney transplant recipient who was admitted approximately 18 months after transplantation with fever, headache, arthralgia, and rapidly progressive deterioration of renal function (Meier et al., 2007). Initial renal biopsy demonstrated severe tubulitis consistent with Banff grade Ib acute T-cell-mediated rejection. Despite high-dose corticosteroid therapy, renal function continued to deteriorate, ultimately requiring hemodialysis. Investigations for CMV, Epstein–Barr virus (EBV), and BK polyomavirus were negative, whereas serological testing demonstrated an acute hantavirus infection, most likely caused by Dobrava–Belgrade virus. Re-evaluation of the biopsy specimen subsequently revealed interstitial inflammatory infiltrates associated with erythrocyte extravasation at the corticomedullary junction, histopathological findings that are characteristic of hantavirus-associated nephritis in HFRS.

From a clinical perspective, this case illustrates how hantavirus infection may closely mimic an episode of acute allograft rejection. Both HFRS and acute rejection may present with overlapping clinical features, including fever, headache, abdominal or lumbar pain, and rapidly worsening renal function. Consequently, renal biopsy is frequently performed to confirm or exclude rejection and to prevent irreversible graft injury. In the reported case, the initial histopathological evaluation identified tubulitis, one of the defining features of acute cellular rejection according to the Banff classification. However, this interpretation proved inconsistent with the subsequent clinical course, which was more compatible with acute hantavirus infection. Only after histopathological re-evaluation were the characteristic features of hantavirus nephritis recognized. Overall, this case highlights the diagnostic difficulties that may arise in kidney transplant recipients and emphasizes the importance of considering hantavirus infection in the differential diagnosis of acute graft dysfunction, particularly when the clinical course is atypical or fails to respond to conventional anti-rejection therapy.

Central nervous system (CNS) involvement in solid organ transplant (SOT) recipients represents an exceptionally rare and poorly documented manifestation of hantavirus infection. Nevertheless, a recent report described an adult kidney transplant recipient who, despite the absence of the typical manifestations of HFRS, such as acute kidney injury or hemorrhagic complications, developed progressive neurological deterioration approximately 6 months after transplantation, characterized by memory impairment, cognitive dysfunction, delirium, aphasia, and limb hypotonia (Qin and Wang, 2024). Following rapid clinical deterioration, cerebrospinal fluid analysis by metagenomic next-generation sequencing (mNGS) identified a high burden of hantavirus-derived sequences, allowing the diagnosis of hantavirus CNS infection. Despite discontinuation of immunosuppressive therapy and initiation of treatment with ribavirin and intravenous immunoglobulins (IVIG), the neurological condition progressed relentlessly to coma and ultimately resulted in the patient’s death.

To our knowledge, this represents the first reported case of hantavirus infection involving the central nervous system in a solid organ transplant recipient and highlights the unique diagnostic challenges posed by this infection in immunocompromised patients. Neurological manifestations are relatively common after transplantation and may result from multiple causes, including drug-induced neurotoxicity, metabolic disturbances, cerebrovascular disease, or opportunistic infections. Furthermore, immunosuppressive therapy may alter the host immune response and attenuate the typical clinical manifestations of hantavirus infection, thereby contributing to delayed diagnosis. In this setting, conventional microbiological investigations and clinical findings may be inconclusive, whereas metagenomic next-generation sequencing (mNGS) may represent a valuable diagnostic tool for the differential diagnosis of acute neurological syndromes in solid organ transplant recipients.

## 6 A wake-up call: The MV *Hondius* outbreak and the evolution of risk assessment in WHO reports

The 2026 outbreak of Andes virus (ANDV) aboard the expedition vessel MV Hondius represented the most significant epidemiological event involving hantaviruses in recent years, bringing these viruses back to the forefront of international scientific attention. The evolution of the outbreak can be reconstructed through the four Disease Outbreak News (DON) reports published by the World Health Organization (WHO), which illustrate how risk assessment progressively evolved as new epidemiological and molecular evidence became available (Istituto Superiore di Sanità, 2026; World Health Organization, 2026b; World Health Organization, 2026c; World H ealth Organization, 2026; World Health Organization, 2026d).

The first WHO report, published on 4 May 2026, described an initially alarming situation. Several passengers developed severe respiratory illness, some of whom died, in a setting characterized by prolonged exposure of passengers and crew members within the confined environment of the vessel. At that stage, both the source of infection and the mode of transmission remained uncertain, and WHO emphasized that person-to-person transmission had to be considered among the possible explanations because Andes virus is the only hantavirus for which human-to-human transmission has been conclusively documented (World Health Organization, 2026b).

Subsequent updates progressively clarified the epidemiological picture. According to the latest WHO assessment, the working hypothesis is that the index case acquired the infection before embarkation through exposure on land, although investigations involving the Argentine and Chilean public health authorities remained ongoing to determine the exact source and circumstances of infection. Based on the available epidemiological evidence, exposure in Chile was considered unlikely because the interval between the individual’s visit and symptom onset exceeded the maximum recognized incubation period. The occurrence of secondary cases was consistent with limited person-to-person transmission facilitated by the unique conditions aboard the vessel, where prolonged close contact among passengers and crew was unavoidable. At the same time, the international repatriation of passengers triggered an extensive multinational surveillance and contact-tracing effort involving numerous national public health authorities coordinated by the WHO (World Health Organization, 2026c; World H ealth Organization, 2026).

The final WHO updates, published on 28 May 2026, enabled a more accurate assessment of the international public health risk. Genomic analyses demonstrated a high degree of genetic similarity among the viral isolates, supporting a common source of infection before embarkation followed by limited secondary human-to-human transmission. Furthermore, the estimated effective reproduction number (Rt = 0.7) indicated that the transmission chain was progressively declining and was unlikely to be sustained within the general population. These findings confirmed that, although Andes virus retains the unique ability among hantaviruses to spread between humans, transmission remains restricted to specific epidemiological settings and does not support a pandemic scenario (World Health Organization, 2026d).

Beyond the epidemiological evolution of the outbreak itself, the WHO reports also documented the rapid international public health response. Through the mechanisms established under the International Health Regulations (IHR), updated diagnostic protocols, case definitions, biosafety procedures, and recommendations for clinical management and contact tracing were rapidly disseminated and subsequently incorporated into the Hantavirus Outbreak Toolbox, developed to support national public health authorities in managing the event (World Health Organization, 2026d; World Health Organization, 2026e).

Of particular importance was the publication of the Interim Guidance for the management of contacts associated with the MV Hondius outbreak. This document introduced operational case and contact definitions specifically tailored to Andes virus and adopted a highly precautionary approach. Relevant exposures included not only direct contact with biological fluids but also prolonged presence in shared enclosed spaces, participation in communal meals, and close proximity to symptomatic individuals. Given the uncertainty surrounding the transmission dynamics at the early stages of the outbreak, all passengers and crew members who had been on board after a predefined date were classified as high-risk contacts and placed under active surveillance for 42 days, corresponding to the maximum recognized incubation period for Andes virus. Surveillance measures included recommendations regarding isolation, travel restrictions, clinical monitoring, and strict biosafety precautions (World Health Organization, 2026f).

Overall, the MV Hondius outbreak did not fundamentally change the biological understanding of hantaviruses. Rather, it provided a compelling demonstration of how international surveillance systems can dynamically refine risk assessment as new epidemiological and molecular evidence emerges. More than signaling the emergence of a new pandemic threat, this event highlighted the importance of a rapid, coordinated, and evidence-based public health response integrating epidemiology, molecular diagnostics, and international surveillance. At the same time, it reinforced the central role of the One Health approach in the preparedness and management of emerging zoonotic diseases and underscored the need for heightened clinical awareness, particularly among vulnerable populations such as solid organ transplant recipients and other immunocompromised patients (World Health Organization, 2026d; World Health Organization, 2026e; World Health Organization, 2026f).

## 7 Discussion and conclusions

Over the past decade, hantaviruses have gained relevance among emerging infectious diseases less because of their limited capacity for human-to-human transmission than because of the ecological dynamics that sustain their circulation. The evidence reviewed here indicates that human risk is governed by the interplay among animal reservoirs, humans, and the environment, with climate change, ecosystem disruption, urbanization, and globalization progressively widening opportunities for spillover. Seen in this way, hantaviruses are best understood not as isolated rodent-borne infections but as a model of environmentally driven zoonotic emergence, one that extends the One Health paradigm toward the broader framework of Planetary Health and makes their ecology a strategic priority for outbreak preparedness.

A recurring theme in the recent literature is the role of companion animals. Dogs and cats are not natural reservoirs; however, preliminary evidence suggests that their exposure may reflect local viral circulation and that they may occasionally introduce infected rodents or contaminated excreta into the home. Pet rats are a more direct concern, having served as reservoirs of Seoul orthohantavirus in documented human outbreaks across Europe and North America. Together these observations argue for integrating veterinary and human surveillance, so that animal monitoring can help identify areas at increased risk of spillover.

Therapeutic options remain limited. Ribavirin is still the only antiviral with demonstrated clinical benefit in humans, and only when given early in HFRS (chiefly Hantaan virus infection), while it confers no convincing benefit in HPS/HCPS; corticosteroids failed to improve outcomes in a randomized trial. Supportive intensive care therefore remains the cornerstone of management. This pattern reflects a disease in which severity is driven not by viral replication alone but by endothelial dysfunction, dysregulated immune activation, and vascular leakage, the same biology that explains ribavirin’s narrow early-phase window and provides the biological rationale for investigating host-directed therapies. However, clinical evidence supporting these approaches remains very limited, and the only host-directed strategy evaluated in a randomized clinical trial, high-dose corticosteroid therapy, failed to demonstrate clinical benefit.

Against this background, direct-acting antivirals, neutralizing monoclonal antibodies, host-directed agents, and DNA vaccines are under active investigation. In particular, monoclonal antibody research remains at an early translational stage, with evidence derived primarily from *in vitro* neutralization studies, structural characterization of conserved epitopes, and protective efficacy in animal challenge models. Although these findings provide a rationale for the development of broadly reactive immunotherapeutic strategies, no monoclonal antibody has yet progressed to advanced clinical trials or demonstrated pan-hantavirus efficacy in humans. Consequently, adequately powered clinical studies are still required before these approaches can be considered for routine clinical use (Ramos-Nino et al., 2026; Clark et al., 2026).

Immunocompromised patients, and solid organ transplant recipients in particular, are a further area of concern. Although the available evidence is currently limited to isolated case reports and does not allow estimation of disease incidence or pretest probability, hantavirus infection may be considered in the differential diagnosis of transplant recipients presenting with unexplained graft dysfunction, acute kidney injury, or neurological deterioration when compatible epidemiological exposure is present. This recommendation is based on clinical plausibility and the potential consequences of delayed diagnosis rather than on quantified risk estimates. Given the continued expansion of transplantation programs and international travel, maintaining a high index of clinical suspicion in the appropriate epidemiological context remains the most appropriate approach.

Finally, the 2026 MV Hondius outbreak was a pivotal contemporary event. Rather than revealing a new biological threat, it showed how international surveillance can refine risk assessment in near-real time by integrating epidemiological, clinical, and genomic data. Successive WHO Disease Outbreak News reports corroborated the existing understanding, based on 3 decades of epidemiological evidence from Chile and Argentina, that although Andes virus is uniquely capable of person-to-person transmission, spread remains restricted to specific epidemiological settings involving close and prolonged contact and does not support sustained community transmission. It is a reminder that a single spillover event can escalate rapidly into an international public health emergency requiring coordinated action. Several limitations apply. Much of the underlying literature comprises observational studies, case reports, animal models, and small cohorts, with few adequately powered randomized trials, and the evidence in transplant recipients is almost entirely anecdotal. Substantial heterogeneity across viral species, geographic settings, clinical syndromes, and study designs limits direct comparison, and, as a narrative review, this work synthesizes the available evidence without a predefined protocol or quantitative meta-analysis.

In conclusion, hantaviruses exemplify emerging zoonoses for which prevention cannot rely on pathogen-specific control alone but must address the interactions among humans, animals, and the environment. Translating current knowledge into practice will require earlier diagnosis, standardized surveillance, and continued development of targeted antiviral, immunomodulatory, and preventive strategies, with One Health operationalized as coordinated surveillance and Planetary Health providing the conceptual basis for confronting the environmental drivers of future emergence (Whitmee et al., 2015). Beyond hantavirus itself, this framework may help clinicians, researchers, and public health authorities anticipate and respond to the next emerging zoonotic threat.
